# Intelligent Analysis Algorithm for Satellite Health under Time-Varying and Extremely High Thermal Loads

**DOI:** 10.3390/e21100983

**Published:** 2019-10-10

**Authors:** En-Hui Li, Yun-Ze Li, Tian-Tian Li, Jia-Xin Li, Zhuang-Zhuang Zhai, Tong Li

**Affiliations:** 1 School of Aeronautic Science and Engineering, Beihang University, Beijing 100191, China; lienhui@buaa.edu.cn (E.-H.L.); litiantian@buaa.edu.cn (T.-T.L.); jxin.lee@buaa.edu.cn (J.-X.L.); 2 Institute of Engineering Thermophysics, North China University of Water Resources and Electric Power, Henan 450045, China; 3 Advanced Research Center of Thermal and New Energy Technologies, Xingtai Polytechnic College, Hebei 054035, China; 4 School of Automation Science and Electrical Engineering, Beihang University, Beijing 100191, China; zhaizz@buaa.edu.cn; 5 Chengyi Academy of PKUHS, Peking University, Beijing 100080, China; litong@i.pkuschool.edu.cn

**Keywords:** satellite, dynamic health evaluation, fuzzy reasoning

## Abstract

This paper presents a dynamic health intelligent evaluation model proposed to analyze the health deterioration of satellites under time-varying and extreme thermal loads. New definitions such as health degree and failure factor and new topological system considering the reliability relationship are proposed to characterize the dynamic performance of health deterioration. The dynamic health intelligent evaluation model used the thermal network method (TNM) and fuzzy reasoning to solve the problem of model missing and non-quantization between temperature and failure probability, and it can quickly evaluate and analyze the dynamic health of satellite through the collaborative processing of continuous event and discrete event. In addition, the temperature controller in the thermal control subsystem (TCM) is the target of thermal damage, and the effects of different heat load amplitude, duty ratio, and cycle on its health deterioration are compared and analyzed.

## 1. Introduction

With the development of satellite space missions, satellite health is facing severe challenges due to the drastic changes of thermal environment and internal thermal load. Due to the change of satellite orbit or transfer [1] or the different working modes of satellite components, time-varying thermal load will be caused. In addition, due to the highly integrated package of electronic equipment [2,3], the use of high heat flux density components [4] and wireless energy transmission, the transient thermal load of the satellite is extremely large. Such as lasers, electronic chips, and advanced propulsion devices, are expected to involve high heat fluxes (above 100 W/cm^2^) [5,6]. Time variations and extremely high thermal loads can affect satellite health and even lead to satellite system failures [7,8]. Therefore, the rapid and effective dynamic health assessment of satellites is of great significance.

According to data from insurance analysis, from 25% to 28% [9,10] of satellite failures on orbit, are related to the electrical power system. The main failure mode of electronic equipment is thermal failure, and its failure rate increases exponentially with increasing temperature [11]. The traditional researches on the influence of temperature on the failure rate of components are based on empirical models, such as the exponential model of Arrhenius [12]. Therefore, there is a lack of accurate quantitative relationship between temperature and failure rate.

The past research on satellite health is to calculate the lifetime and reliability of satellites through reliability analysis. Traditional reliability analysis is based on probabilistic statistical analysis of large amounts of in-orbit data. Therefore, to study the satellite fault caused by thermal failure requires necessary data to determine various parameters of the model. Unfortunately, limited empirical data and statistical analysis of satellite reliability exist in the technical literature [13]. Many methodologies such as failure mode effect analysis (FMEA) and the fault tree analysis (FTA) are used in the reliability analysis [14,15]. However, this model has some limitations in reliability analysis. It is not easy for these models to conduct further quantitative analysis automatically due to the lack of effective means of mathematical expression [16]. In addition, the traditional reliability analysis model only considers failure death or survival and lacks analysis methods for sub-health.

In order to analyze the satellite thermal health response under time-varying and extreme thermal loads, an intelligent evaluation model based on fuzzy logic is proposed. In addition, this paper presents an evaluation index that can represent satellite sub-health state. Fuzzy reasoning is used to solve the problem of missing models and non-quantization which are caused by temperature. The continuous process simulation and discrete event simulation of thermal load change complete efficient collaborative computation by thermal health assessment algorithm. Finally, the effects of typical thermal loads on satellite health are analyzed.

## 2. Dynamic Health Intelligent Evaluation Model

In order to analyze the health deterioration of satellites under time-varying and extreme thermal loads, the dynamic health intelligent evaluation model (DHIEM) is presented in this paper. Meanwhile, new definitions such as health degree and failure factor and new topological system (satellite-subsystems-components) considering the reliability relationship are proposed to characterize the dynamic performance of health deterioration. This section introduces the principle and algorithm of the model in detail.

### 2.1. Principle Description and Evaluation Index

The DHIEM is combined with the TNM. TNM solves the dynamic temperature distribution of the satellite based on thermal load, and DHIEM can dynamically evaluate the thermal health index of the satellite based on the output of TNM. So, this subsection first introduces the three definitions used in the model.

The failure probability of the component is obtained according to its dependence on temperature, as shown in formula 1. Then, according to the failure probability of the component, the health degree and failure factor of the subsystems can be obtained. Similarly, according to the health degree of the subsystems, the health degree and failure factor of the satellite can be calculated, as shown in Equations (2)–(3).

#### 2.1.1. Definition of Evaluation Index

- Failure Probability ξ(t):

The failure probability refers to the instantaneous probability of failure of the components when the components run to a certain time. The failure probability value is affected by temperature and satisfies the equation:

(1) ξ(t)=f(T,dT/dt)

where, ξ(t) is the failure probability value at a certain time; T and dT/dt are the temperature and its difference of the moment respectively; Function relation f is fuzzy inference.

- Health Degree H(t):

Health degree is to evaluate the health of the whole system through the statistics of the damage of each component of the system. Health degree refers to the percentage of the number of units in the system that are not damaged at the current moment when the system runs to a certain time t.

(2) $$H(t)=1-\frac{N_{failure}}{N_{total}}$$

where, $N_{total}$ is the total number of the underlying component units constituting the system, and $N_{failure}$ is the number of failure failures of the underlying component units in the system at the current moment.

- Failure Factor F(t):

Failure factor describes the deterioration speed of system failure. Failure factor refers to the ratio of the increment of failure sub-units in the system within unit time t to the number of healthy component units at time t.

(3) $$F(t)=\frac{N_{failure}(t+\Delta t)-N_{failure}(t)}{\Delta t\cdot [N_{total}-N_{failure}(t)]}$$

where, $N_{failure}(t+\Delta t)-N_{failure}(t)$ is the number of newly added fault failure unit in time; $N_{total}-N_{failure}(t)$ is the number of remaining underlying component units, that is, the number of component units that have not failed by time t; Δt is the time interval taken.

#### 2.1.2. Principle Description

**Figure 1:**
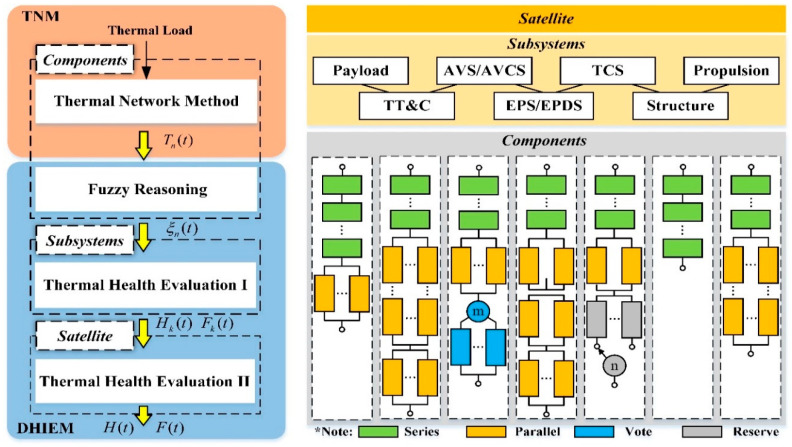
The principle of satellite dynamic health intelligent evaluation model: (**a**) Model schematic; (**b**) Topological system (satellite-subsystems-components).

The principle of satellite dynamic health intelligent evaluation model is shown in Figure 1. First, the dynamic temperature $T_{n}(t)$ of satellite component n under different thermal load conditions can be calculated by using the TNM. Considering the influence of temperature and its difference on the failure probability of satellite components, fuzzy reasoning is used to quantitatively analyze the failure probability $\xi_{n}(t)$ of components. Then, the health degree $H_{k}(t)$ and failure factor $F_{k}(t)$ of subsystem k are obtained by using the thermal health evaluation I to cooperative solve continuous process simulation and discrete event simulation. Finally, the thermal health evaluation II is used to calculate the satellite’s health degree H(t) and failure factor F(t) according to the subsystem’s health degree $H_{k}(t)$.

The model divides the satellite into satellite-subsystems-components topological system according to its functional composition. In general, a satellite consists of a payload and the common subsystems supporting the payload. Typical public subsystems include structural subsystem, thermal control subsystem (TCS), energy/distribution subsystem, telemetry tracking and command subsystem (TT&C), attitude/orbit control subsystem and propulsion subsystem [17]. Each subsystem is composed of different functional components. The topological system takes into account the series, parallel, vote and reserve relations of various components of the satellite, so as to reflect the influence of component failure on subsystems or satellite health more accurately.

Section 2.2 uses the thermal network model to calculate the dynamic temperature distribution of components. In Section 2.3, the relationship between the failure probability of the component and the temperature of the component is analyzed quantitatively by fuzzy inference. Section 2.4 introduces a dynamic evaluation algorithm that calculates the health degree and failure factor of subsystems and satellite by analyzing the failure of components.

### 2.2. Component Temperature Dynamic Modeling

In order to study the effects of thermal damage on satellite health, it is necessary to calculate the dynamic distribution of satellite temperature. The thermal network model is used to solve the temperature distribution of satellite. In the satellite thermal network model, each equipment component is treated as an isothermal body and a node, and the node temperature represents the average temperature of the isothermal body. It is in itself an approximate method because of the discretization needed to solve the heat transfer differential equation. However, due to simplicity and agility, lumped parametric models are more and more widely used in satellite thermal analysis.

The TNM is described in detail elsewhere [18,19]. For any satellite component (node) j, the non-linear algebraic transient heat exchange equations that are obtained and that have to be solved is expressed by Equation (4).

(4) $${\left(cm\right)}_{j}\frac{dT_{j}}{dt}=Q_{sj}+Q_{pj}+\sum_{i}{\left(\frac{A_{c}\lambda }{\delta }\right)}_{i,j}(T_{j}-T_{i})+\sum_{i}{\left(\varepsilon A_{r}\right)}_{i,j}\sigma (T_{j}^{4}-T_{i}^{4})$$

where, $Q_{sj}$ represents the external heat absorbed by node j; $Q_{pj}$ represents the heat that it is directly produced in the j node itself. Any satellite component (node) has heat exchange with other nodes through heat conduction and heat radiation. $\sum_{i}{\left(\frac{A_{c}\lambda }{\delta }\right)}_{i,j}(T_{j}-T_{i})$ represents the thermal conduction heat transfer between node j and the rest of the nodes of the model. $A_{c}$ is the thermal conductivity area, λ is the thermal conductivity coefficient, and δ is the thermal conductivity thickness. The subscript indicates that the three parameters corresponding to the heat conduction and exchange between different nodes have different values.$\sum_{i}{\left(\varepsilon A_{r}\right)}_{i,j}\sigma (T_{j}^{4}-T_{i}^{4})$ represents the radiation heat transfer between node j and the rest of the nodes of the model.ε is the emissivity of the node, $A_{r}$ is the radiation area of the node, and σ is the Stefan-Boltzmann constant. The relative positions of different nodes must also be considered in calculating the radiative heat transfer between them. ${\left(cm\right)}_{j}$ is the heat capacity of node j. The external heat flux $Q_{sj}$ varies with the position and spatial attitude of the satellite in orbit, which is a function of time. The thermal power $Q_{pj}$ may also be a function of time, depending on what the component needs to accomplish.

### 2.3. Component Failure Probability Fuzzy Modeling

In order to quantitatively analyze the influence of component temperature and change on its failure probability, an intelligent calculation model based on fuzzy reasoning is proposed. The failure probability analysis based on fuzzy logic solves the law analysis of the influence of temperature factor on the failure probability of components and completes the qualitative to quantitative analysis and calculation. As a kind of "grey box" system, fuzzy reasoning table has the characteristics of short development cycle, nonlinearity and no need to establish mathematical model, but its difficulty lies in the acquisition of fuzzy rules [20,21].

#### 2.3.1. Fuzzy Reasoning

Fuzzy reasoning goes through three basic processes: fuzziness, fuzzy reasoning, and clearness. Fuzzification—compare input variables and membership functions to obtain membership values of each language identifier; Fuzzy reasoning—performs union operation (usually multiplication or minimization) on the membership function of the initial part to obtain the activation right of each rule, and relies on the activation right to produce the effective result of each rule; Clarity—overlay all valid results to produce a clear output.

**Figure 2:**
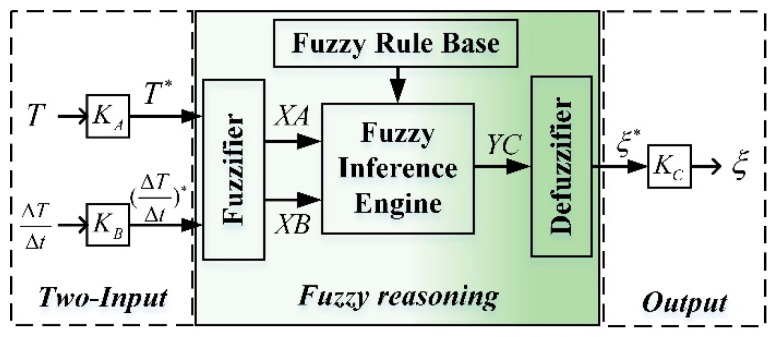
Fuzzy reasoning system.

The failure probability of unit is related not only to temperature but also to the rate of change of unit time temperature. Therefore, the intelligent evaluation model algorithm proposed in this paper adopts the fuzzy reasoning decision structure with double input and single output. The double inputs are the temperature and the rate of change of temperature per unit time, and the output is the failure probability of component units. The fuzzy reasoning decision system is shown in Figure 2.

Figure 2 shows the fuzzy reasoning system consisting of a fuzzifier, a fuzzy inference engine, a defuzzifier, and a fuzzy rule base. The double inputs to the fuzzifier are the temperature $T^{*}$ and its difference ${\left(\frac{\Delta T}{\Delta t}\right)}^{*}$ normalized by the factors $K_{A}$ and $K_{B}$. Considering that the actual variation range of the theoretical domain of different component elements may be different, in order to normalize the model, we uniformly adopt the standardized theoretical domain [−1, 1] and use different transformation factors to transform the practical theoretical domain. Similarly, the output ξ scaled by the factor $K_{c}$. The transformation factor satisfies the following equation:

$$\{\begin{array}{c} T^{*}=K_{A}\left(T-\frac{T_{\max}+T_{\min}}{2}\right)\\ {\left(dT/dt\right)}^{*}=K_{B}\left[\left(dT/dt\right)-\frac{{\left(dT/dt\right)}_{\max}+{\left(dT/dt\right)}_{\min}}{2}\right] \end{array}$$

(5) $$\{\begin{array}{c} K_{A}=\frac{2}{T_{\max}-T_{\min}}\\ K_{B}=\frac{2}{{\left(dT/dt\right)}_{\max}-{\left(dT/dt\right)}_{\min}} \end{array}$$

**Figure 3:**
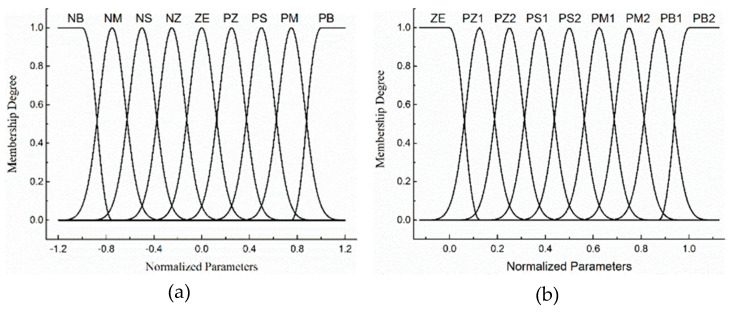
Membership degree functions: (**a**) input; (**b**) output.

The input and output variables to the fuzzy reasoning system are characterized by the fuzzy sets, linguistic values, and associated analytical ranks shown in Table 1. In order to facilitate the formulation of fuzzy rule table, each fuzzy set is divided into 9 element levels. The subscript of the fuzzy set represents which element level the language value is related to. Each fuzzy set is defined by a Gaussian membership function shown in Figure 3. The membership functions have an overlap with each other to provide a smooth output transition between the regions. The input of fuzzy logic (temperature, temperature change rate per unit time) is not completely attributed to a certain class (fuzzy set). That is to say, there is no strict boundary between higher and higher temperatures, and fuzzy theory uses membership degree to measure the degree of attribution of variables to each set. The process of transforming the input of logic into the membership of each fuzzy set is called fuzzification.

The failure probability of a component is not only related to temperature, but also related to electrical stress, mechanical stress, fatigue and other factors. Some component failure probabilities are mainly affected by temperature, while some component temperatures have less influence on their failure probability. Therefore, the specific dependence of the failure probability of components of different functions on temperature determines the range of failure probability of the actual component. We also use the standardization domain for generalization transformation. Considering the non-negative nature of failure probability, the corresponding failure probability uses the standardization domain [0, 1], its fuzzy set and fuzzy language as Table 1(b).

The fuzzy reasoning output is determined using the linguistic rules in the following form:

$$\mathrm{IF}\;T^{*}\;\mathrm{is}\;XA_{i}\;\mathrm{and}\;{\left(\frac{\Delta T}{\Delta t}\right)}^{*}\;\mathrm{is}\;XB_{j},\;\mathrm{THEN}\;\xi^{*}\;\mathrm{is}\;YC_{l(i,j)}$$

$\mathrm{where}XA_{i},XB_{j},\mathrm{and}YC_{l(i,j)}$ are the fuzzy sets reflecting the linguistic values of $T^{*}$, ${\left(\frac{\Delta T}{\Delta t}\right)}^{*}$, and $\xi^{*}$, respectively, and the subscript variables *i*, *j*, and (*i*, *j*) denote the analytical rank associated with the linguistic values in a nine-element set defined in Table 1.

Defuzzification is the inverse of fuzziness. The function of clearness is to transform the fuzzy quantity obtained by fuzzy reasoning into the actual clearness and adopt the clearness method given by the following.

(6) $$\xi^{*}=\frac{\sum_{i=1}^{9}\sum_{j=1}^{9}\xi^{*}{}_{l(i,j)}\mu_{l(i,j)}}{\sum_{i=1}^{9}\sum_{j=1}^{9}\mu_{l(i,j)}}$$

In (6), $\xi^{*}{}_{l(i,j)}$ and $\mu_{l(i,j)}$ are the discrete element and membership degree of the output fuzzy set $YC_{l(i,j)}$ representatively.

#### 2.3.2. Fuzzy Rule Design

Realize the influence of temperature and unit time temperature on the failure probability of components. In the environment of drastic temperature change, the material and composite parts will be damaged such as fracture and avulsion. Temperature and thermal deformation due to temperature gradient will lead to fatigue failure. When the temperature cycle of components changes more than ±20 ℃, their failure efficiency can be increased to more than 8 times that of the basic constant temperature. In the case of a constant temperature, no matter whether the rate of temperature change per unit time is positive or negative, the greater the relative change, the more drastic the temperature change is, the greater the impact on the failure probability. 

**Figure 4:**
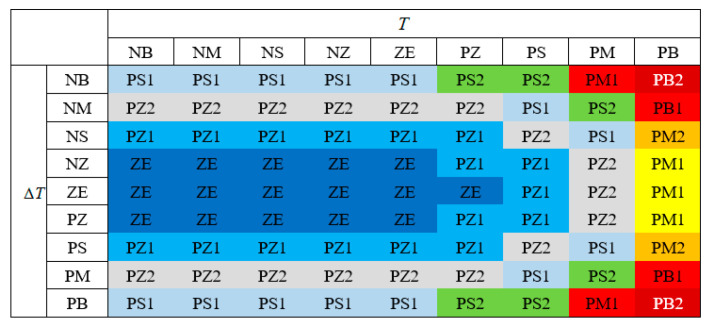
Fuzzy rule I.

In the fuzzy set with double input of temperature and temperature change rate per unit time, the grade subscripts corresponding to different fuzzy language values meet the following criteria. Then, Equation 7 can be used to generate the fuzzy rule table as shown in Figure 4.

(7) $$Z(i,j)=\mathrm{Round}[(a\times e^{bi})+c\times j^{2})]$$

The most commonly used Arrhenius model to describe the reaction rate is Jacobus. The equation shows that the failure rate increases exponentially with the increase of temperature. However, very low temperatures also have a significant effect on failure probability. For example, low temperatures can cause some materials to break and become brittle. Therefore, the low temperature case, though not as big as the high temperature effect, should also be considered.

**Figure 5:**
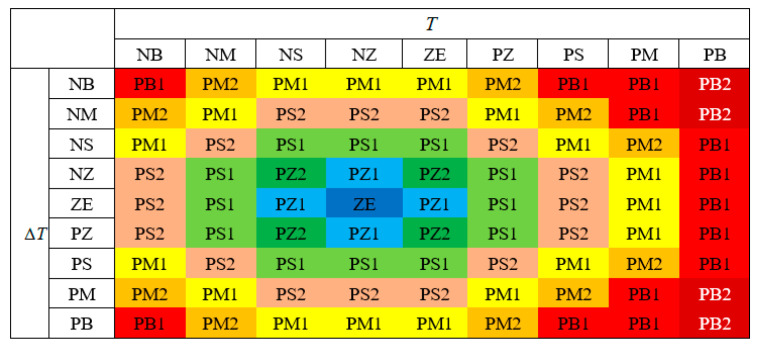
Fuzzy rule II.

When considering the influence of low temperature on failure probability, in the fuzzy set with double-input temperature and temperature change rate per unit time, the grade subscripts corresponding to different fuzzy language values and their corresponding failure rates meet the following criteria. Then, equation 8 can be used to generate the fuzzy rule table as shown in Figure 5.

(8) $$Z(i,j)=\mathrm{Round}{\left[a\times {\left(i+b\right)}^{2}+j^{2}\right)}^{0.5}]$$

### 2.4. System Health Dynamic Evaluation Modeling

**Figure 6:**
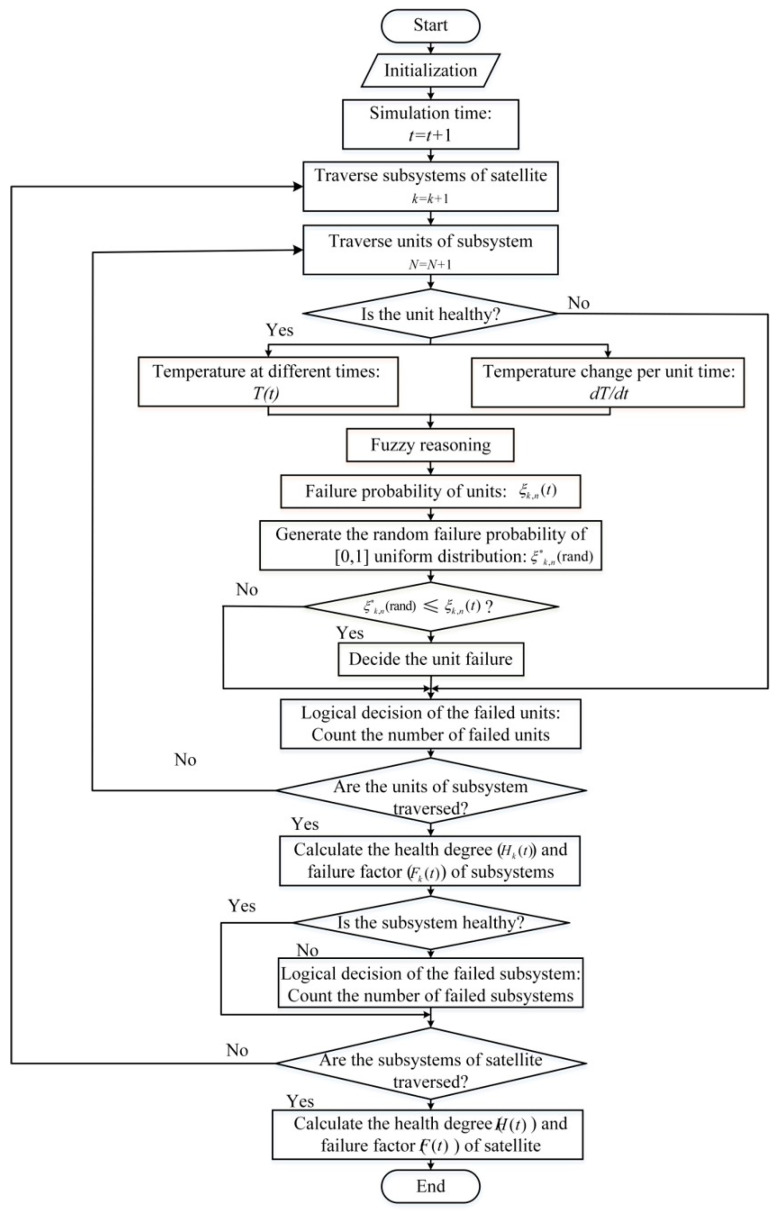
Algorithm process.

Detailed dynamic iterative calculation simulation flow chart is shown in the Figure 6. First, initialize the parameters required to set up the model. Specifically, it includes fuzzy segmentation in fuzzy reasoning and parameter setting of membership function, setting of conversion factor and design of fuzzy rules; and the simulation calculates the clock zero, generates the seed initialization settings for the random number. To characterize the randomness of a component’s failure at each moment, we use a pseudo-random number to produce a uniformly distributed failure rate. The component is judged to be invalid by comparing the random failure rate of the component with the failure rate calculated by the fuzzy inference. Then, the health and failure factors of the subsystem or system are evaluated by counting the number of failed components at each moment.

There are several key points: 

(1) After the simulation starts, in each simulation time, it is traversed in order according to the topology of the whole star -> subsystem -> component unit;

(2) Each time the component unit of the subsystem is traversed, it is judged whether the unit is healthy, because once the unit is determined to be unhealthy at the last moment, it is not judged at the current moment;

(3) Each non-failed component unit correspondingly generates a random probability of random distribution of [0, 1], and compares with the random failure probability calculated by the component unit, thereby determining whether the component unit fails at the current moment;

(4) The logical determination method of the failed component is that if it is a tandem type component unit, as long as the failure occurs, its health deterioration effect on the subsystem composed of the unit is counted. However, the parallel type unit and the voting unit type reserve type unit do not malfunction when one system fails. Therefore, for the latter three types, the number of failed component units is counted only when the number of failures has an impact on the deterioration of the health state of the system.

## 3. Results and Discussion

### 3.1. Cases Design

This section will mainly describe the normal operation condition of the satellite in orbit and the damage conditions under different thermal loads. During the normal operation of the satellite in orbit, the satellite’s thermal control system makes each component of the satellite work within its own normal temperature range, so the satellite’s health will not change significantly in the short term. However, it should be noted that the time-varying and extremely high thermal loads coupled with the full sun/eclipse operating environment yield large temperature changes and large thermal gradients, which will seriously affect the satellite’s health and life. So, we designed four groups of thermal load damage conditions that could reflect different time-varying characteristics to study the health changes of satellite typical subsystems.

#### 3.1.1. Parameters Setting for Normal Orbital Operation

The external heat flux (solar radiation heat flux, earth albedo heat flux and earth infrared radiation heat flux) of the satellite in each direction (± X, ± Y, ± Z) varies periodically during the normal orbit cycle. The satellite orbit parameters used in the simulation analysis are shown in Table 2. The simulation time is one orbital period (1.63 h), in which, the initial moment of the entire simulation cycle is the time when the satellite-Z direction is subjected to the maximum solar radiation intensity. The satellite entered the earth’s shadow at 1884.97 s and left the earth’s shadow at 3968.28 s.

The external heat flow is calculated using the software THERMAL DESKTOP based on the orbital parameters of the satellite. Then, by importing the calculated external heat flow results into THIEM, the dynamic temperature of each component node of the satellite can be solved, and the health index of the satellite in normal orbit can be obtained.

#### 3.1.2. Design of Thermal Damage Conditions

In order to analyze the effects of thermal damage caused by time-varying and extreme thermal loads on satellite health during orbit operation, we designed four conditions as shown in Table 3. In these cases, case I serves as a reference case. By comparing the Cases II, III, IV and the reference case I, respectively, the effects of the magnitude, duty cycle and period of different thermal loads on satellite health can be analyzed. The pulse rectangular wave is used in the time-varying heat load. Where duty ratio refers to the proportion of thermal load time relative to the total time in an impulse cycle. For example, in Case I, the duty ratio is 1/6, indicating that the thermal load of 10 s is 2 W/cm^2^ in an impulse cycle, and the remaining heat load of 50 s is 0 W/cm^2^.

The target of thermal damage is the temperature controller in the thermal control subsystem. The temperature controller as a thermal control component guarantees the normal operation of other satellite core components, but it cannot withstand large thermal loads because it does not have good cooling. So, the final simulation selects 2 W/cm^2^ and 4 W/cm^2^. In the initial moment of simulation, the target temperature controller is loaded with extreme thermal loads and the satellite thermal health response is solved by using the THIEM.

### 3.2. Effects of Thermal Load Amplitude on Satellite Health

**Figure 7:**
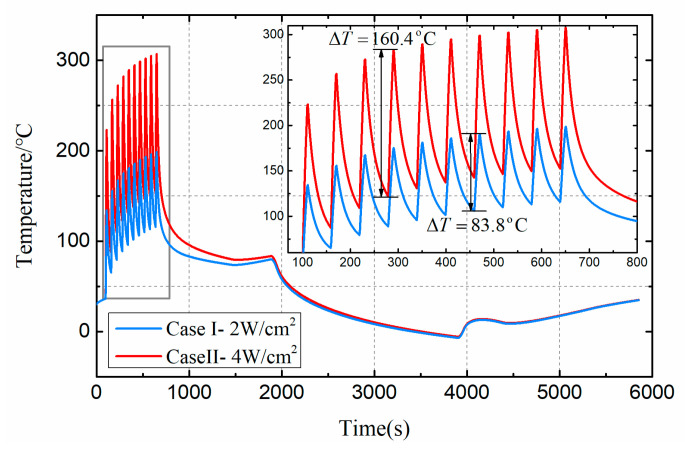
Dynamic temperature of damaged components under Case I and Case II.

Figure 7 shows the effects of different thermal load amplitudes on the dynamic temperature of target damaged component. Under normal orbit operation, the temperature range of the temperature controller is 0 to 50. However, under the action of the pulse thermal load, the temperature of the target damaged component rose rapidly and changed periodically. After 10 cycles of thermal loading, the temperature of the damaged component increased cumulatively. This is because the component cooling time caused by the thermal load of 0 is short, resulting in insufficient cooling, which eventually leads to a cumulative increase in temperature. Comparing Case I and Case II, the temperature rise caused by different thermal load amplitude was variant in one operating period. This is because the temperature rise of the same component in the same time interval is positively correlated with the thermal load.

**Figure 8:**
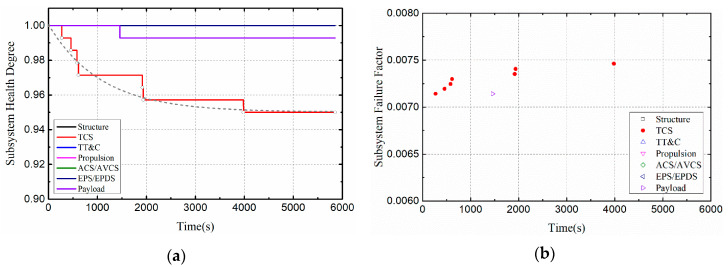
Health indicators of satellite subsystems (Case I): (**a**) Health degree. (**b**) Failure factor.

**Figure 9:**
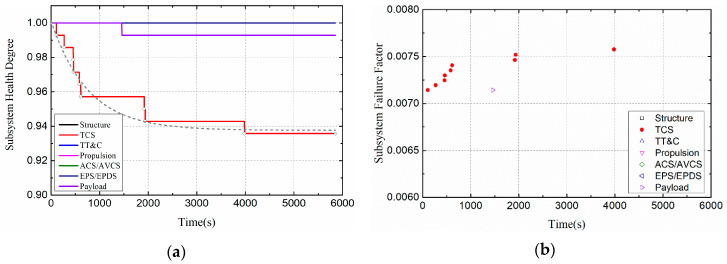
Health indicators of satellite subsystems (Case II): (**a**) Health degree. (**b**) Failure factor.

When the heat flux density is 2 W/cm^2^ and 4 W/cm^2^ respectively, the health degree and failure factors of the satellite subsystems are shown in Figure 8 and Figure 9. As shown in Figure 8a and Figure 9a, the health degree of the satellite’s thermal control system is exponentially decreasing, and the health degree of the satellite’s payload is also slightly damaged. This is because the thermal damage of the temperature controller component will directly affect the health of the thermal control subsystem. As mentioned earlier, the temperature controller is designed to serve the payload, so when the controller is damaged, the payload health is also affected. As shown in Figure 8b and Figure 9b, the failure factor of the satellite thermal control subsystem increased. According to the definition of failure factor, failure factor is not equal to 0, which means that a component will fail at that time. Therefore, the distribution of the failure factor can determine the speed of failure.

**Figure 10:**
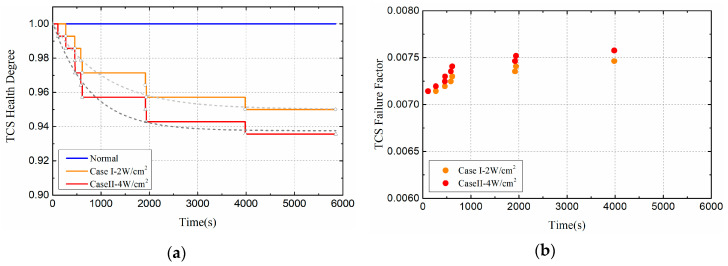
Health indicators of thermal control subsystem (TCS) (Compare Case I and Case II): (**a**) Health degree. (**b**) Failure factor.

In order to visually compare and analyze the effects of different thermal load amplitudes on satellite systems, this section mainly discusses the health changes of thermal control subsystems, as shown in Figure 10. Compared with the normal orbital condition, the health of the thermal control system decreased with time, indicating that the health of the subsystem is deteriorated to some extent. Moreover, as the heat flux density increased, the health condition of the thermal control subsystem deteriorated more severely. In Case I, the health degree of the TCS eventually dropped to 0.95; in Case II, the health degree of the TCS eventually fell to 0.936. Moreover, the greater the thermal load, the earlier the system is damaged. This is because the higher the heat load, the higher the temperature of the damaged component, which makes the component more vulnerable to failure.

### 3.3. Effects of Thermal Load Duty Ratio on Satellite Health

**Figure 11:**
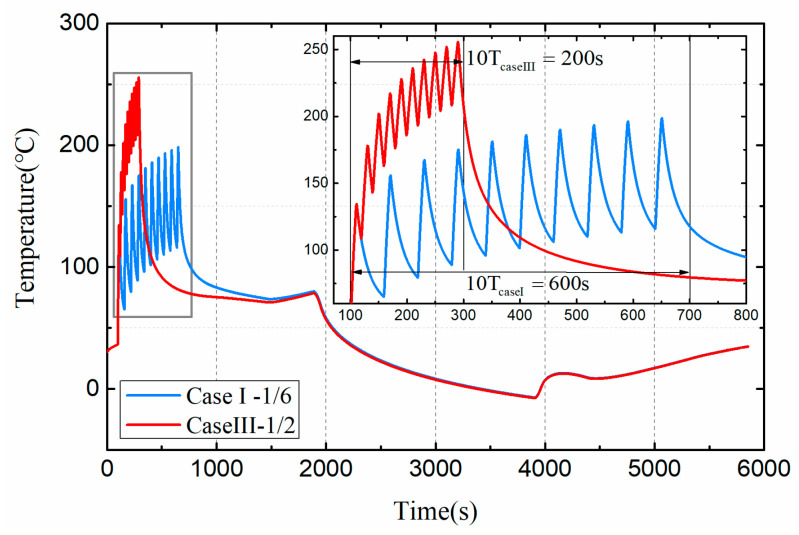
Dynamic temperature of damaged components under Case I and Case III.

Figure 11 shows the effect of different heat load duty cycles on the dynamic temperature of the target damage component. In both cases, the thermal load amplitude and cycle number are the same, and the loading time of thermal load in one cycle is the same, which is 10 s. The duty ratio of pulsed thermal load directly affects the cooling time of a cycle, and finally leads to the difference of the total acting time of thermal load. The total acting time of pulse of case I is 600 s, while the total acting time of pulse of case III is 200 s. However, compared to case I, Case III causes the temperature of damaged components to rise more rapidly and eventually reach a higher temperature. This is because the pulse duty ratio is small, resulting in insufficient cooling of the damaged components and eventually continuous accumulation of temperature.

**Figure 12:**
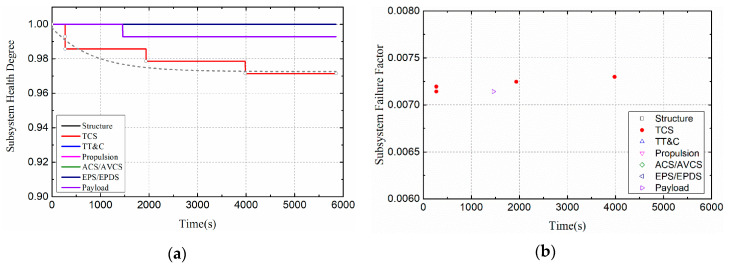
Health indicators of satellite subsystems (Case III). (**a**) Health degree. (**b**) Failure factor.

**Figure 13:**
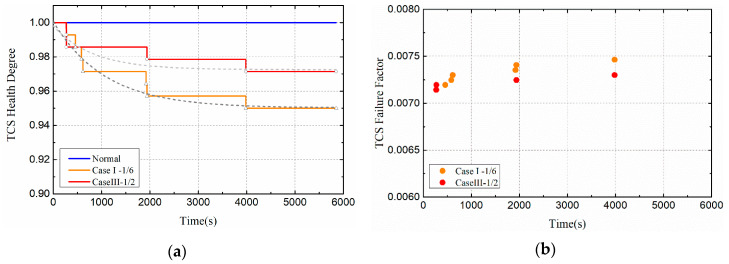
Health indicators of TCS (Compare Case I and Case III). (**a**)Health degree. (**b**)Failure factor.

Figure 12 shows the changes of satellite system health indicators when the duty cycle of thermal load is 1/2. The health degree of the satellite thermal control subsystem decreased exponentially to 0.97. In order to intuitively compare and analyze the impact of thermal load duty ratio on the health of satellite subsystem, this section shows the health impact of Case I and Case III on the thermal control subsystem in Figure 13. As shown in Figure 13a, when the duty ratio of thermal load is 1/2, the health degree of the thermal control subsystem decreased to 0.97, while when the duty ratio of thermal load is 1/6, and the health degree of the thermal control subsystem decreased to 0.95. Although the higher the thermal load duty ratio will lead to the higher temperature rise of the damaged components, the total loading time will be reduced. In general, components have the ability to respond to extreme environments in the short term, so short-term high temperatures do not necessarily lead to complete failure. As shown in Figure 13b, the higher the duty cycle of the thermal load causes the thermal control subsystem to fail earlier. In addition, it can also be found from the fit curve of health in Figure 13a that the slope of Case III is larger than that of Case I. This is because the heating time in a cycle is the same, the greater the thermal load duty ratio, the more drastic the temperature change of the damaged components.

### 3.4. Effects of Thermal Load Cycle on Satellite Health

**Figure 14:**
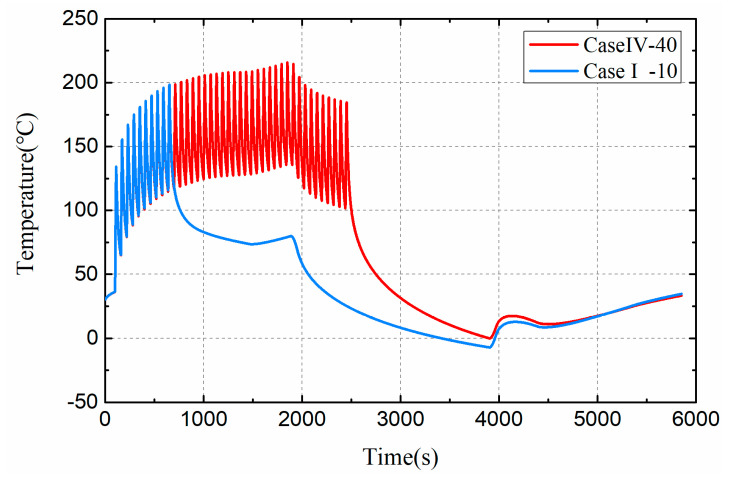
Dynamic temperature of damaged components under Case I and Case IV.

Figure 14 shows the effect of different heat load cycles on the dynamic temperature of the target damage component. The number of thermal load cycles directly affects the loading time of thermal load. Under the action of thermal load cycle, the cumulative temperature of damaged parts rises to about 220℃. The pulsed thermal load causes an increase in the temperature of the satellite components, and the longer the loading time results in a higher temperature rise of the damaged component. In Case IV, the temperature of damaged component dropped after 1884 s. This is because the satellite entered earth’s shadow in 1884 s and became colder.

**Figure 15:**
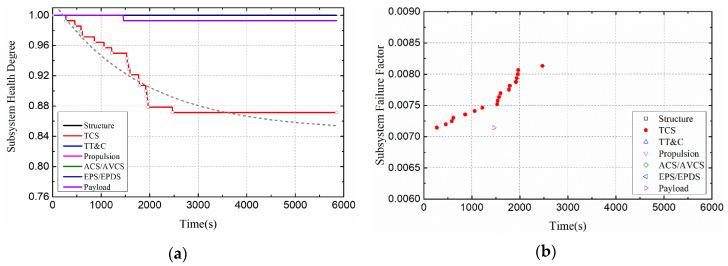
Health indicators of satellite subsystems (Case IV). (**a**) Health degree. (**b**) Failure factor.

**Figure 16:**
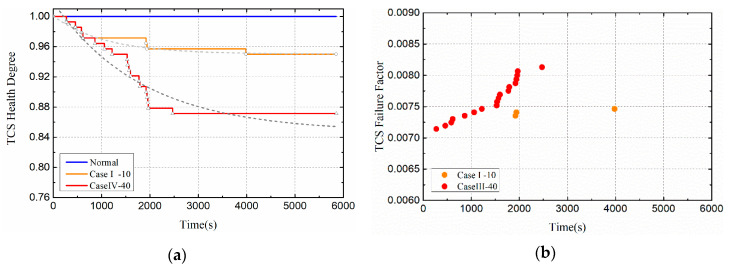
Health indicators of TCS (Compare Case I and Case IV). (**a**)Health degree. (**b**)Failure factor.

Figure 15 shows the changes of satellite system health indicators when the cycle of thermal load is 40. The health degree of the satellite thermal control subsystem decreased exponentially to 0.87. In order to intuitively compare and analyze the impact of thermal load cycles on the health of satellite subsystem, this section shows the health impact of Case I and Case IV on the thermal control subsystem in Figure 16. As shown in Figure 16a, when the cycle of thermal load is 40, the health degree of the thermal control subsystem decreased to 0.87, while when the cycle of thermal load is 10, and the health degree of the thermal control subsystem decreased to 0.95. With the increase of thermal load cycle, the deterioration of thermal control subsystem becomes more serious.

These results show that the health of the components in the satellite subsystem is affected by time-varying and extreme thermal loads. The analysis of cases accords with the qualitative law of theory.

## 4. Conclusions

This paper presents an intelligent evaluation model based on fuzzy logic for satellite thermal health analysis. A new evaluation index and topological system are proposed to evaluate satellite health status. Taking the temperature controller in the satellite thermal control subsystem as the target of thermal load, the health deterioration of the satellite under typical working conditions such as time-varying and maximum thermal load is analyzed. Based on the analytical investigations presented in this paper, the following conclusions may be made:

1. The fuzziness of the relationship between temperature and failure probability is considered, and the relationship between temperature and failure probability is quantitatively described by intelligent analysis method (fuzzy reasoning).
2. The model can quickly and accurately evaluate the effects of different thermal conditions on satellite health. The health deterioration of the system is characterized by the change of health degree and failure factor.
3. Multi-period, high heat flux density, and low duty ratio have great influence on satellite health.

The model solves the problem of collaborative solution of different models (satellite thermal analysis is continuous process simulation, satellite component failure is discrete probability event simulation), as well as the challenges of model absence and non-quantification in satellite thermal health analysis. The model can better reflect the dynamic deterioration process of the system health. In the future, it can be transported in many systems with strict life requirements, and only the topology system needs to be modified according to different systems.

## Figures and Tables

**Table 1:** Fuzzy sets and linguistic values: (**a**) input; (**b**) output.

(a)			(b)		
**Fuzzy Sets**	**Ranks**	**Linguistic Values**	**Fuzzy Sets**	**Ranks**	**Linguistic Values**
NB	−4	Negative big	ZE	0	Zero
NM	−3	Negative medium	PZ1	1	Positive zero 1
NS	−2	Negative small	PZ2	2	Positive zero 2
NZ	−1	Negative zero	PS1	3	Positive small 1
ZE	0	Zero	PS2	4	Positive small 2
PZ	1	Positive zero	PM1	5	Positive medium 1
PS	2	Positive small	PM2	6	Positive medium 2
PM	3	Positive medium	PB1	7	Positive big 1
PB	4	Positive big	PB2	8	Positive big 2

**Table 2:** Satellite orbit parameters.

Parameters	Value
Solar incident angle	17.23 °C
Orbit altitude	641.65 km
Average of solar radiation	1354 W/m^2^
Albedo	0.35
Earth infrared radiation	221.484 W/m^2^
Space temperature	4 K

**Table 3:** Thermal damage conditions and value.

Cases	Amplitude	Duty Ratio	Cycle
I	2 W/cm^2^	1/6 (Heat 10 s; Cool 50 s)	10
II	4 W/cm^2^	1/6 (Heat 10 s; Cool 50 s)	10
III	2 W/cm^2^	1/2 (Heat 10 s; Cool 10 s)	10
IV	2 W/cm^2^	1/6 (Heat 10 s; Cool 50 s)	40
